# Compositional Variation in *Trans*-Ferulic, *p*-coumaric, and Diferulic Acids Levels Among Kernels of Modern and Traditional Maize (*Zea mays* L.) Hybrids

**DOI:** 10.3389/fnut.2020.600747

**Published:** 2020-12-22

**Authors:** Mariana Zavala-López, Sherry Flint-García, Silverio García-Lara

**Affiliations:** ^1^Tecnologico de Monterrey, School of Engineering and Science, Monterrey, Mexico; ^2^Agricultural Research Service, U.S. Department of Agriculture, Columbia, MO, United States

**Keywords:** maize, diversity, landrace, NAM, hybrids, phenolics

## Abstract

Maize is one of the most heterogenous cereals worldwide in terms of yield, physical characteristics, and biochemical composition due to its natural diversity. Nowadays the use of maize hybrids is extensive, while the use of landraces is mostly local. Both have become an important genetic resource useful to identify or generate varieties with desirable characteristics to overcome challenges of agronomic performance, nutritional quality, and functionality. In terms of functionality, one of the most studied families of compounds are phenolic acids. These compounds have been associated with the improvement of human health because of their antioxidant capacity. To evaluate the diversity of phenolic compounds in maize, two collections, the Nested Association Mapping (NAM) founders and 24 landraces, were crossed with B73. Phenolic compounds were extracted and quantified by HPLC-PDA. Soluble and cell wall phenolic acids were identified and significant differences between and within the NAM and Landrace collections were assessed. Soluble *p*-coumaric acid quantification of B73 × NAM hybrids presented high variation as the range went from 14.45 to 132.34 μg/ g dw. In the case of B73 × Landrace hybrids, wide variation was also found, ranging 25.77–120.80 μg/g dw. For *trans*-ferulic acid, significant variation was found in both hybrid groups: B73 × NAM presented an average of 157.44 μg/g dw (61.02–411.13 μg/g dw) whereas the B73 × Landrace hybrids average was 138.02 μg/g dw (49.32–476.28 μg/g dw). In cell wall *p*-coumaric acid, a range from 30.93 to 83.69 μg/g dw and 45.06 to 94.98 μg/g dw was found for landrace and NAM hybrids, respectively. For cell wall *trans*-ferulic acid, a range from 1,641.47 to 2,737.38 μg/g dw and 826.07 to 2,536.40 μg/g dw was observed for landrace and NAM hybrids, respectively. Significant differences between hybrid groups were found in *p*-coumaric acid, for both soluble and cell wall-bounded. Therefore, maize hybrids produced by conventional techniques using both modern and traditional varieties showed a high diversity in terms of phenolic compounds, denoting the role of these compounds in the maize ability to endure different environment conditions. This study provides a platform of comparison through the unveiling of maize phenolic compounds for future breeding efforts.

## Introduction

Maize has been positioned as one of the most important crops worldwide, with an annual production of over 1 million tons in the US (1). It is also a staple crop for developing countries like Mexico and large regions in Africa (2). The rate of population growth and climate change has given the food and agricultural industry the new task to not only produce large quantities of food, but also with better nutritional quality and functionality (3). One of the available sources to achieve this challenge is the genetic diversity of maize landraces (4). A maize landrace can be defined as a population of a cultivated plants with defined historical origin, distinct identity, and lack of formal crop improvement, often showing genetic heterogeneity, are generally adapted to specific regions by traditional farming systems (5). Maize natural diversity makes the identification of landraces a continuous activity, as they are an important genomic resource to revisit existing varieties, and discover new desirable characteristics or to generate modern hybrids. At least 59 distinct landraces have been recognized in Mexico alone (6).

Since maize was domesticated, human population overgrowth has defined the way in which plant breeding approaches the fulfillment of food supply required (2). B73 is the public inbred line that serves as the reference genome, it dates to 1972, and was the original parent of many commercial North American hybrids (7). Furthermore, it has been suggested that commercial North American germplasm available today is based on only seven inbred lines, including B73 (8). An evaluation of maize genetic contribution in the USA showed that B73 has over 126 descendants in the present commercial germplasm (9). Nowadays, modern hybrids are developed to address different agronomic challenges; increased yield (10), higher protein content (11), drought resistance (12), and pest resistance (13) are just some examples. An extensive collection of recombinant inbred lines has been developed, called the Nested Association Mapping (NAM) population (14). NAM is a genetic resource for the analysis of quantitative traits in maize that combines the two most representative approaches for this type of analysis: linkage mapping and association analysis (15) but little is known about phenolic acid composition in this population.

Phenolic compounds are an important group of secondary metabolites widely distributed in plants that are associated with the improvement of human health because of their antioxidant capacity. Phenolic compounds have been studied before in maize, and higher concentrations of these compounds have been found in maize compared to other cereals (16). Maize has two primary phenolic compounds, *p*-coumaric and *trans*-ferulic acids, mainly related with cell wall bound phenolic acids (17). Diferulic acids have been also reported and associated with maize resistance to pests (18), and the presence of phenolic amides has been recently reported in a core collection of 32 Mexican maize landraces (19). Although phenolic compounds have been previously studied in maize, an evaluation of their variation in a wide collection of varieties such as NAM and a broader set of landraces would help to identify genotypes with potential for breeding these traits.

To evaluate the natural variation of phenolic compounds of maize hybrids, in both landraces and modern inbreds, two collections (the NAM founders and 24 landrace inbreds) were crossed with B73. Phenolic compounds were extracted and quantified by HPLC-PDA. Soluble and cell wall bound phenolic acids were identified, differences between and within NAM and Landrace collections were assessed, and the effect of the geographic origin was determined.

## Materials and Methods

### Biological Materials

The landrace materials used in this study included 4 agroecological origin groups: tropical/subtropical, temperate, northern flint and mixed. Tropical/subtropical were represented by MR01 (Araguito), MR03 (Bolita), MR04 (Canilla), MR05 (Cateto), MR07 (Comiteco), MR08 (Costeno), MR09 (Cravo Riogranense), MR10 (Crystalino Norteno), MR11 (Cuban Flint), MR16 (Pepetilla), MR18 (Reventador), MR21 (Tabloncillo), MR22 (Tuxpeno), MR23 (Zapalote Chico), MR25 (Poropo), and MR26 (Pollo); temperate by MR13 (Hickory King), and MR20 (Shoe Peg); northern flint by MR19 (Santo Domingo), MR14 (Longfellow Flint), MR02 (Assiniboine), and MR12 (Havasupai); and mixed by MR06 (Chapalote), MR15 (Palomero de Jalisco). These landrace inbred lines were produced by self-pollination from open-pollinated landraces accessions as described by Chia et al. (20) and Hufford et al. (21) and are referred to hereafter as LR.

The modern inbred lines used were the parental lines for the NAM population (referred to hereafter as NAM) and classified by population structure with genetic marker data according to Flint-Garcia et al. (22). The materials were classified in the following agro-ecological categories: tropical/subtropical, temperate, northern flint and mixed. The tropical/subtropical was represented by NC350, CML103, CML333, Tzi8, Ki11, Ki3, CML69, NC358, CML228, CML247, CML52, CML322, and CML277, the non-stiff stalk temperate group by MS71, Oh43, B97, Ky21, M162W, and Oh7B, the northern flint group by P39, Il14H, and HP301, and the mixed group by Mo18W, M37W, and Tx303.

### Production of Hybrids and Field Design

The common parent for hybrid production was the line B73 inbred. Production was performed over four different seasons: 24 and 2 entries were produced in Columbia, MO and Puerto Rico, USA, in 2008, respectively, 18 entries were produced in Puerto Rico in 2009; and 6 entries were produced in 2010 in Columbia, MO, USA. Hybrids were produced by controlled hand-pollination using B73 as female. Then, seed materials used for the grain analysis were generated in Aurora, NY, USA, during 2012. A randomized complete block design with 3 m × 0.9 m rows in three replicates was used for planting the 25 B73 × NAM founder hybrids (to generate the NAM group) and 24 B73 × landrace hybrids (to generate the LR group). For each row, three to five plants were self-pollinated. The ears were hand-harvested and dried to 12–13% moisture, bulk-shelled, and were stored at 4°C before shipping. Grain samples were ground on dry ice and shipped at −20°C to Bio-Sciences Laboratory at Tecnologico de Monterrey (Mexico) for further processing and analysis. Each sample (20 g) was homogenized by grind milling (mesh <1 mm), and homogenized samples were stored at −20°C until phenolic composition analysis was done.

### Phenolic Compounds Extraction

Ground samples of the LR and NAM collections were used for the extraction of phenolic compounds as described previously (23). Briefly, 80% methanol was used to extract soluble phenolic compounds in triplicate for each hybrid growth in the field (50 mg). The remaining pellet was subjected to alkaline hydrolysis for 1 h and neutralized. Two hexane washes were performed to remove lipids and three ethyl-acetate washes were applied to recover cell-wall bound phenolic compounds. Cell wall bound phenolic extracts were dried with gaseous nitrogen and dissolved in 50% methanol. All the extracts were stored at −20°C until their analysis.

### Quantification of Phenolic Compounds

Extracted phenolic compounds were analyzed by the method of Ayala-Soto et al. (24) and adjusted by Zavala-López and García-Lara (23) using a HPLC system (Agilent 1100 Santa Clara, CA) coupled with a photodiode array (PDA) detector (Agilent G1315D, Santa Clara, CA). Linear gradient elution was performed using HPLC-grade water (CAS: 7732-18-5, BDH, West Chester, PA) acidified (pH = 2) with trifluoroacetic acid (CAS: 76-05-1, Sigma-Aldrich, St. Louis, MO) and acetonitrile (CAS: 75-05-8, BDH, West Chester, PA), at a flow rate of 0.6 mL/min at 25°C. Phenolic compounds were separated with a Zorbax SB-Aq, 4.6 mm ID × 150 mm (3.5 um) reverse phase column. The Chemstation software (for LC Copyright© Agilent Technologies, 1990–2003) was used to process the data and command the equipment. Peak identification of *trans*-ferulic acid (CAS: 537-98-4, Sigma-Aldrich, St. Louis, MO) and *p*-coumaric acid (CAS: 501-98-4, Sigma-Aldrich, St. Louis, MO) was based on retention time and absorption spectra of these standards. Identification of diferulic acids was performed according to retention times and absorption spectra reported by Ayala-Soto et al. (24) and expressed as equivalents of ferulic acid.

### Statistical Analysis

Phenolic acids were extracted and quantified by triplicate for each sample. Analysis of variance (α = 0.05) was used to identify significant differences between genotypes for both groups of hybrids using Minitab 18.1® Software (2017). Significant differences found with ANOVA were further analyzed by least significant difference (LSD, α = 0.05) to identify specific differences among groups of hybrids. The main effects and interactions effects of the geographic origin, or type of hybrid were also evaluated.

## Results

### Soluble Phenolic Acids

**Figure 1:**
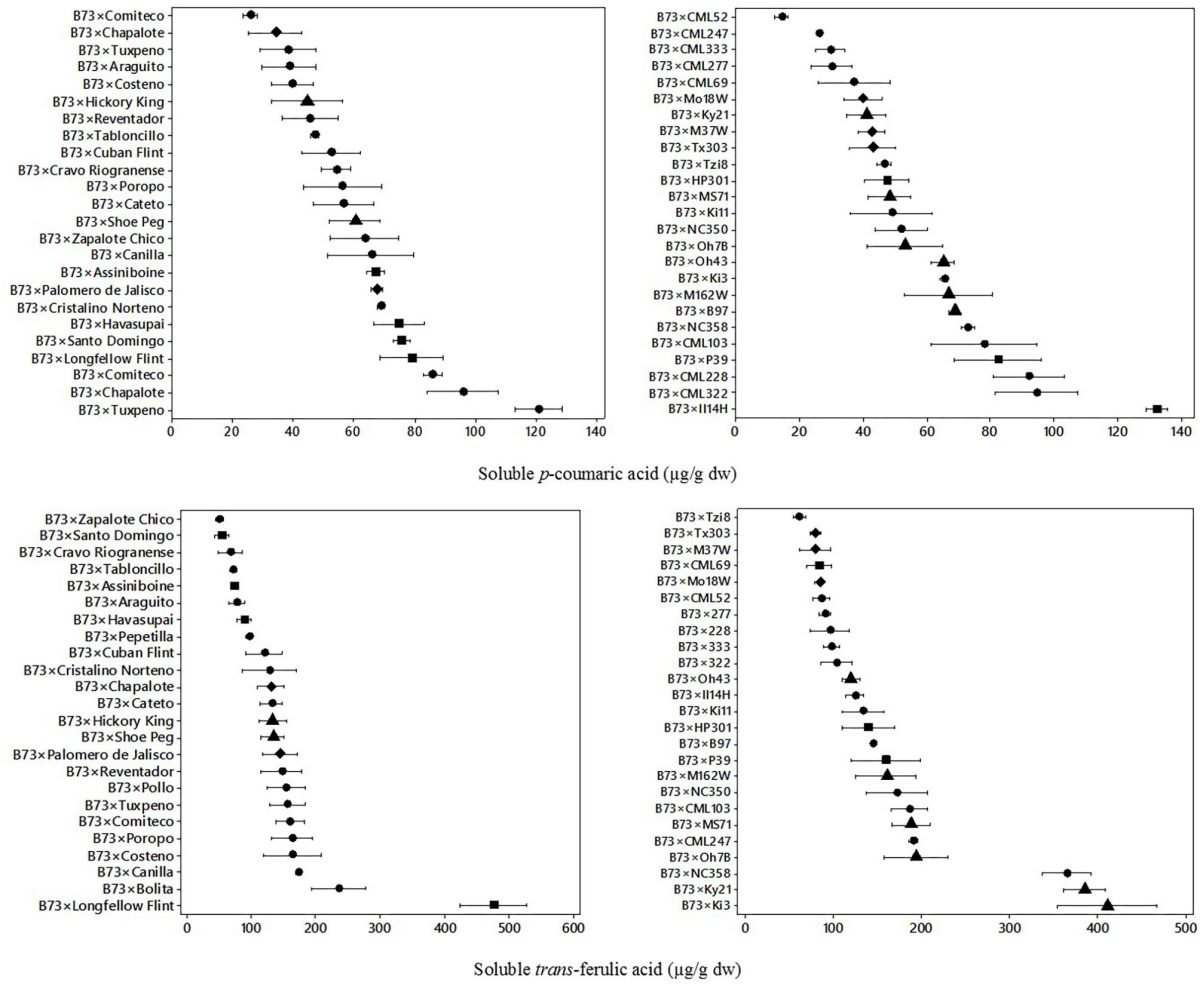
Means and variability ranges of soluble *p*-coumaric acid (upper) and soluble *trans*-ferulic acid (lower) in B73 × landrace hybrids (left) and B73 × NAM hybrids (right). Different figures denote different origin; 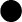 tropical/subtropical, 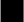 northern flint, 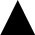 temperate, and 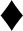 diamond: mixed. Concentration in μg/g dw: μg per gram of dry weight.

Phenolic acids of B73 × Landraces and B73 × NAM hybrids were analyzed to evaluate their variation. Figure 1 shows the dispersion of the soluble phenolic acids in B73 × Landraces and B73 × NAM hybrids for soluble *p*-coumaric acid and *trans* ferulic acid.

In landrace hybrids, a wide variation was observed in almost all genotypes evaluated. The mean soluble *p*-coumaric acid content in landrace hybrids was 61 μg/g dw, with a range of 26–121 μg/g dw. In NAM hybrids, the mean soluble *p*-coumaric acid was 57 μg/g dw, with a range of 14–132 μg/g dw. A wide variation of *p*-coumaric acid between genotypes was found for the landrace hybrids, in a similar dispersion range as the one found in NAM hybrids. A six-fold difference was found between the lowest and highest hybrids in both landrace and NAM hybrid sets.

A significant effect (*p* = 0.001) from genotype, type of hybrid (landrace vs. NAM), and geographic origin and hybrid^*^origin was found for soluble *p*-coumaric acid (Table 1). Both types of hybrids (landrace and NAM) with northern flint origin had higher contents of soluble *p*-coumaric acid (>78 μg/g dw). In contrast, hybrids from mixed origin had lower soluble *p*-coumaric acid content (<45 μg/g dw). The interaction of the main effects was also evaluated, resulting in hybrid type × geographic origin had a significant effect. Landrace hybrids from the northern flint had an overall higher content of soluble *p*-coumaric acid (>90 μg/g dw).

**Table 1:** Analysis of variance of main effects and interaction for principal soluble and cell wall phenolic acids in kernels of modern and traditional maize hybrids.

**Source**	**Degrees of freedom**	**Soluble *p-*coumaric acid**	**Soluble *trans*-ferulic acid**	**Cell-wall bound *p*-coumaric acid**	**Cell-wall bound *trans*-ferulic acid**	**5–5^**′**^ DFA**	**8-O-4 DFA**	**Total DFAs**
Genotype	24	2,706^***^	53,304^***^	1,115^*^	408,951^*^	113^*^	1,465^**^	5,651^***^
Origin	3	4,578 ^***^	25,762^*^	2,860^**^	278,846	20	2,036^**^	454
Hybrid	1	4,777 ^***^	26,871^*^	2,481^**^	67,135	32 ^*^	1,419^**^	62,568^***^
Hybrid^*^Origin	3	4,397^***^	56,295^***^	117	680,872	345	4,391	2,866
CV		45.7	56.6	39.2	23.2	20.2	21.9	62.57

Origin: tropical/subtropical, northern flint, temperate, and mixed.

Hybrids: B73 × landraces hybrids and B73 × NAM.

*** p ≤ 0.001;

** p ≤ 0.01;

* p ≤ 0.05.

In Figure 1, the dispersion of soluble *trans*-ferulic acid for the landrace hybrids and NAM hybrids is also shown. The mean *trans*-ferulic acid content for the landrace group was 138 μg/g dw, with a range of 49.3–476.3 μg/g dw, and 10-fold difference between the lowest and highest hybrid. For the NAM hybrid group, the mean soluble *trans*-ferulic acid content was 157 μg/g dw and the values ranged from 61.0 to 411.1 μg/g dw. A wider variation, compared with *p*-coumaric acid, was found for soluble *trans*-ferulic acid between different hybrids.

The main effects and the interactions were evaluated for soluble *trans*-ferulic acid (Table 1). In this case, the genotype (*p* = 0.001), the type of hybrid (landrace vs. NAM), and the geographic origin all had a significant effect (*p* = 0.05), where the hybrids from landraces as well as hybrids with temperate origin had higher contents (>220 μg/g dw) overall. The interaction of the hybrid and the geographic origin also had a significant effect (*p* = 0.001). Overall, the NAM hybrids from temperate origin had the highest soluble *trans*-ferulic acid content (>240 μg/g dw).

### Cell Wall Bound Phenolic Acids

**Figure 2:**
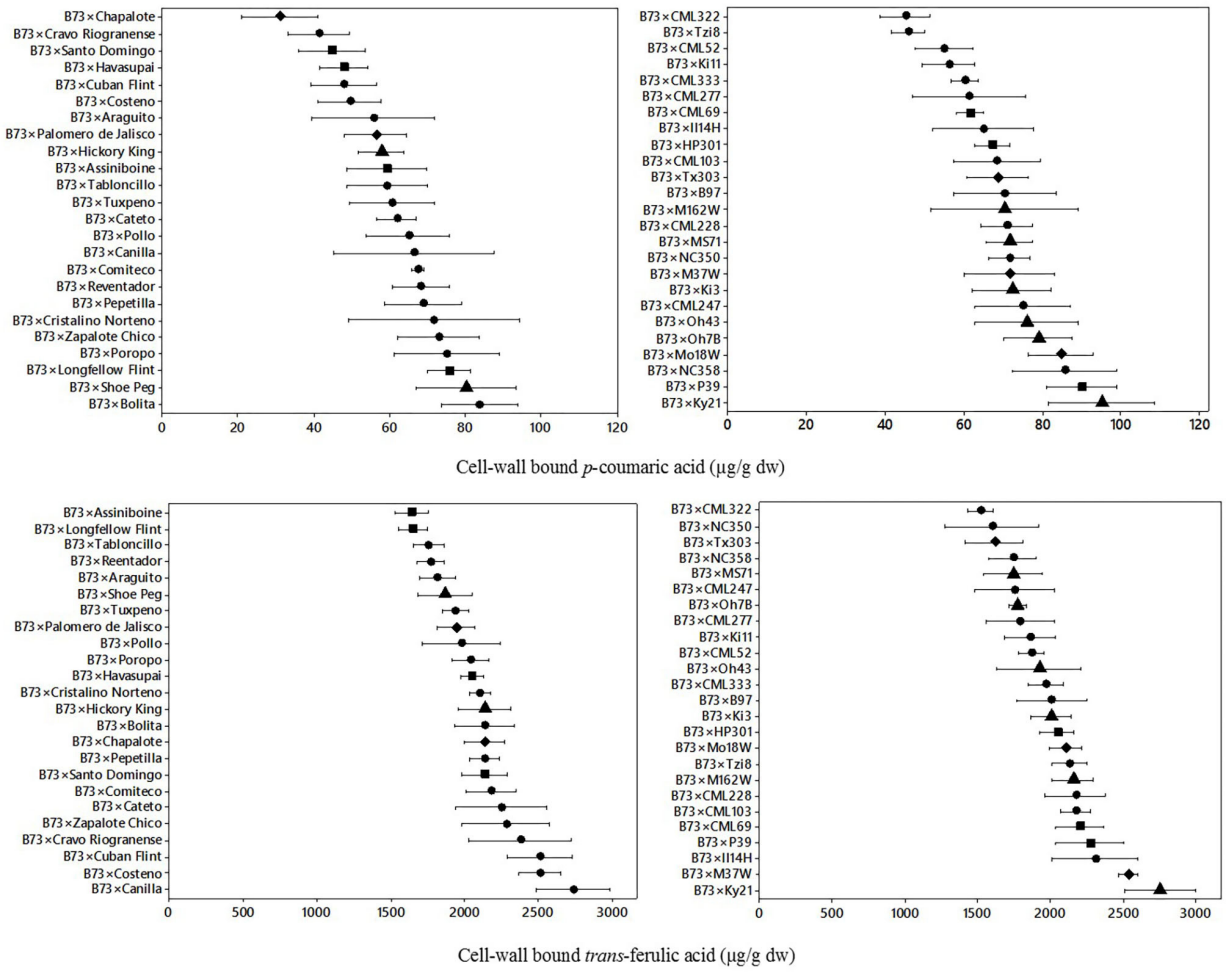
Means and variability ranges of cell-wall bound *p*-coumaric acid (upper) and s cell-wall *trans*-ferulic acid (lower) of B73 × Landraces hybrids (left) and B73 × NAM hybrids (right). Different figures denote different origin; 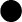 tropical/subtropical, 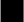 northern flint, 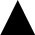 temperate, and 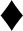 diamond: mixed. Concentration in μg/g dw: μg per gram of dry weight.

The variation of cell-wall bound *p*-coumaric acid and *trans* ferulic acid for B73 × Landrace hybrids and B73 × NAM hybrids is shown in Figure 2. For the landrace hybrids, there was a wider range of bound *p*-coumaric content than its soluble form for most of the hybrids. The mean bound *p*-coumaric acid content was 61.1 μg/g dw with a range of 30.9–83.6 μg/g dw. For the NAM hybrids, the mean bound *p*-coumaric acid content was 69.5 μg/g dw with a range of 45.0–95.2 μg/g dw. Different ranges of bound *p*-coumaric acid were found for both landrace hybrids and NAM hybrids, with the first group presenting a higher range of dispersion. Furthermore, both hybrid groups showed approximately two-fold differences from the lowest to highest content of bound *p*-coumaric acid.

For cell-wall *p*-coumaric acid content, a significant difference effect (*p* = 0.01) was found between the type of hybrid (Table 1), where the NAM hybrids had higher bound *p*-coumaric acid content (>74 μg/g dw). There was also significant variation by genotype (*p* = 0.05) and geographic origin, the highest content of bound *p*-coumaric acid was found in NAM hybrids from mixed and temperate origin (>74 μg/g dw), while the lowest was found on landrace hybrids from mixed origin (<45 μg/g dw).

The dispersion of cell-wall bound *trans*-ferulic acid content for landrace hybrids and NAM hybrids is also shown in Figure 2. In *trans*-ferulic acid of landrace hybrids, the mean content was 2,086 μg/g dw with a range of 1,641–2,737 μg/g dw. For the NAM hybrids, the average was 2,001 μg/g dw with a range of 1,517–2,753 μg/g dw. In this case similar ranges between and within the B73 × Landrace hybrids and B73 × NAM hybrids were found. Based on the analysis of variance (Table 1), there was a significant variation by genotype (*p* = 0.05), but the interaction of hybrid and origin was not significant, even though landrace hybrids from the Northern flint origin and tropical hybrids from the LR group have the higher content.

### Cell Wall Diferulic Acids

**Figure 3:**
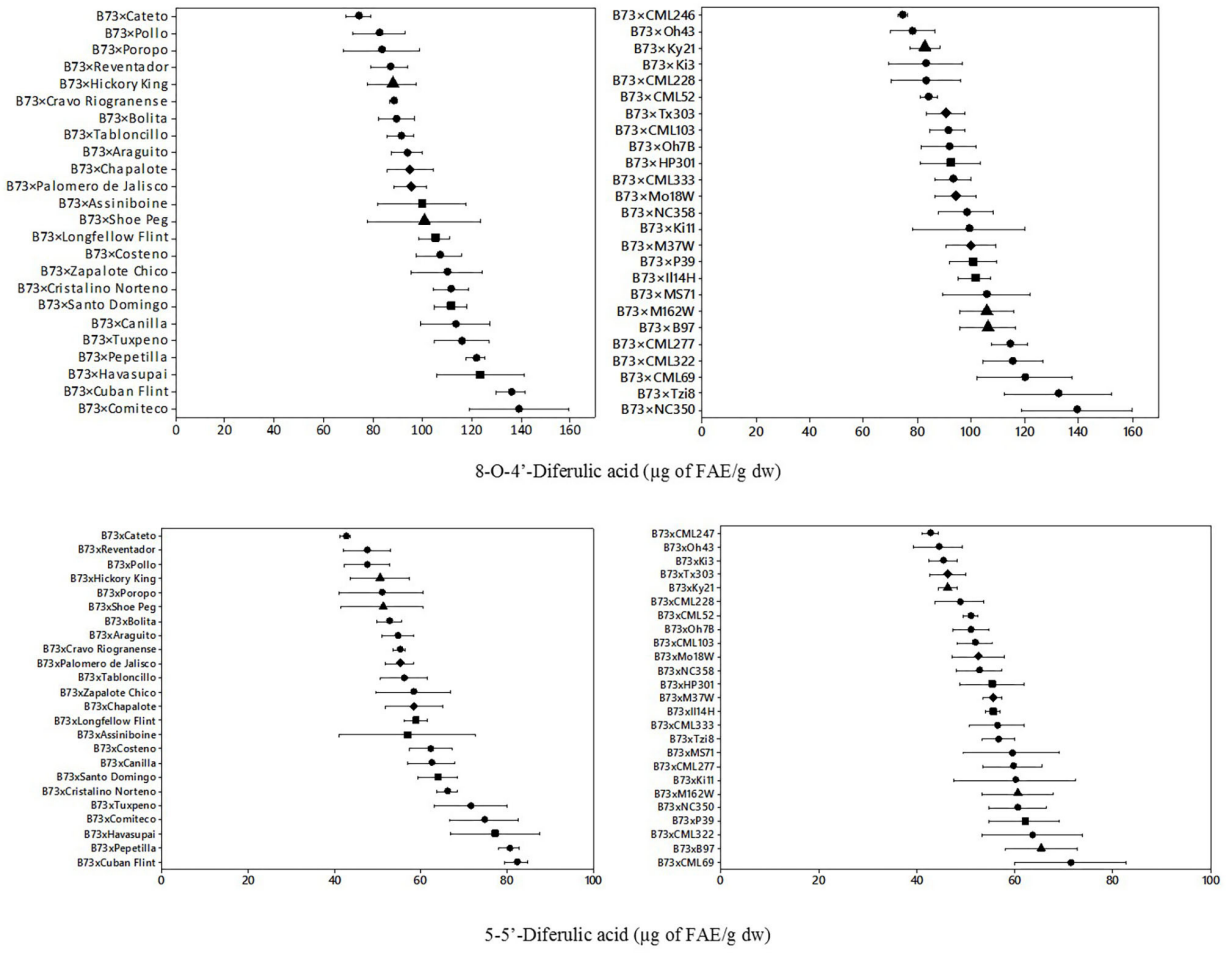
Means and ranges of cell wall 8-O-4′-diferulic acid (upper) and s cell-wall of 5–5′-diferulic acid (lower) in B73 × Landraces hybrids (left) and B73 × NAM (right). Different figures denote different origin; 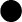 tropical/subtropical, 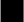 northern flint, 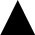 temperate, and 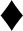 diamond: mixed. Concentration in μg of FAE/g dw: μg of *trans*-ferulic acid equivalents per gram of dry weight.

The quantification of cell wall diferulic acids, specifically 5,5′-diferulic acid (5,5′-DFA) and 8-O-4′-diferulic acid (8-O-4′- DFA), was also performed for both groups of hybrids (Figure 3). Mean content for bound diferulic acids of landrace hybrids were 60.6 μg FAE/g dw for 5,5′-DFA and 103.5 μg FAE/g dw for 8-O-4′- DFA. Two-fold variation was observed in landrace hybrids for 5,5′-DFA (42.55–82.26 μg FAE/g dw), and for 8-O-4′ diferulic acid (74.06–139.20 μg FAE/g dw). Mean content for bound diferulic acids of the NAM hybrids were 54.9 μg FAE/g dw for 5,5′-DFA and 98.9 μg FAE/g dw for 8-O-4′-DFA. Similar ranges of variation were observed for 5,5′-DFA (42.6–71.4 μg FAE/g dw) and for 8-O-4′-DFA (74.3–139.1 μg FAE/g dw). Although similar ranges were found for 5,5′-DFA and 8-O-4 DFA, the landrace hybrids group range was wider than the NAM hybrids group.

For the diferulic acids analyzed (Table 1), type of hybrid and geographic origin had significant main effects for diferulic acid 8-O-4′-DFA, having the highest content the landraces hybrids (>102 μg FAE/g dw) and north flint (>106 μg FAE/g dw). The significant interaction of type of hybrid showed that the highest content was found in landrace hybrids for 5,5′-DFA (*p* = 0.001) and total DFA (*p* = 0.05).

### Analysis of Correlation Between Phenolic Acids

Pearson correlation analysis in NAM and Landrace hybrids was used to establish relationships between main phenolic acids measured in the study (Table 2). Considering both NAM and Landrace hybrids, soluble *p*-coumaric acid was positively correlated (*r* = 0.466; *p* = 0.001) with soluble *trans*-ferulic acid. This correlation was persistent when comparison was performed within the NAM (*p* = 0.05) or Landrace hybrid groups (*p* = 0.001). Additionally, a positive correlation between soluble *trans*-ferulic acid and cell-wall *p*-coumaric acid (*r* = 0.252, *p* = 0.01) and between cell wall *trans*-ferulic acid and cell-wall *p*-coumaric acid (*r* = 0.256, *p* = 0.01) were observed only in the NAM hybrid.

**Table 2:** Pearson correlation between the principal soluble and cell wall phenolic acids found in B73 × Landraces and B73 × NAM hybrids.

	**All Hybrids**			**NAM hybrids**			**Landrace hybrids**		
	**Soluble *p-*coumaric acid**	**Soluble *trans*-ferulic acid**	**Cell-wall bound *p*-coumaric acid**	**Soluble *p-*coumaric acid**	**Soluble *trans*-ferulic acid**	**Cell-wall bound *p*-coumaric acid**	**Soluble *p-*coumaric acid**	**Soluble *trans*-ferulic acid**	**Cell-wall bound *p*-coumaric acid**
Soluble *trans*-ferulic acid	0.467^***^			0.191^*^			0.467^***^		
Cell-wall bound *p*-coumaric acid	0.084	0.091		−0.065	0.252^**^		0.084	0.091	
Cell-wall bound *trans*-ferulic acid	0.035	0.019	−0.126	0.105	0.144	0.256^**^	0.035	0.019	−0.126

*** p ≤ 0.001;

** p ≤ 0.01;

* p ≤ 0.05.

## Discussion

### Soluble Phenolic Acids in Maize Kernel

Recent studies reported that maize kernels have a different content of phenolic compounds in each of their anatomical structures, with the pericarp and aleurone layer having the higher content (25). A large variation between genotypes was found in *p*-coumaric acid for the landrace hybrids, in a similar dispersion range as the NAM hybrids. Phenolic acids have been associated with resistance to different pests, particularly, low levels of *p*-coumaric acid positively correlated to ear rotting (26). However, the evaluation of *p*-coumaric acid in its soluble form is not usually performed (27–31) as the major soluble phenolic compound in maize is *trans*-ferulic acid, although variable ranges are observed between 101 and 236 μg/g dw (32). This study shows that *p*-coumaric acid in its soluble form could potentially aid in the improvement of resistance to biotic stresses and should be included in future analyses for its evaluation. It is interesting that the tropical hybrids in both sets have generally lower *p*-coumaric acid content than the temperate and northern flint hybrids; it is well known that tropical germplasm tends to be more insect and disease resistant than temperate and northern flint types [e.g., (33)] and has been a long-standing focus of the Germplasm Enhancement of Maize Project (34).

A wider variation was found for soluble *trans*-ferulic acid between different hybrids and within some hybrids in both the NAM and Landrace groups. Mora-Rochin et al. (29), evaluated the soluble *trans*-ferulic acid content of different maize varieties and reported a range of 3.4–5.8 μg/g dw for yellow and white maize hybrids, which is well below our findings. In another study with yellow and white maize hybrids, a similar range of 4.4–5.16 μg/g dw was found (35). In hybrids derived by crossing a genetically modified parent with landraces, ranges were observed between 55.6 and 123.6 μg/g dw (32). However, further studies of specialty maize have found a higher value of soluble *trans*-ferulic acid in these varieties, especially in sweet corn (35, 36). Furthermore, purple landraces showed wide ranges of soluble *trans*-ferulic acid, ranging from below the detection limit to 538 μg/g dw (37). In our study the color of the hybrids analyzed ranged from white to orange; no purple varieties were included. It is important to note that each maize kernel of this study comes from selfed ears of hybrids, so every kernel in a sample is segregating for millions of SNPs (Single nucleotide polymorphisms) between B73 and the other parent (20). B73 is a yellow endosperm maize with colorless pericarp (yellow endosperm/colorless pericarp); thus selfed samples of B73 crossed with a white parent would be segregating for kernel color and any compounds associated with pericarp composition in our study. Furthermore, B73 and the other parent differ for genes underlying phenolic acids, suggesting a population derived from B73 and one of these “high variation” parents would be good genetic resource for mapping the quantitative trait loci (QTL) including the phenolic acids as has been previously shown (38, 39). Nevertheless, the wide diversity of genotypes in both hybrids collections which was key for their original selection could explain the wide range of soluble *trans*-ferulic acid not previously reported.

### Cell Wall Bound Phenolic Acids in Maize Kernel

High compositional variation of cell-wall bound *p*-coumaric acid were found for both landrace hybrids and NAM hybrids, with the first group presenting a higher range of dispersion. Both ranges fall outside the OECD established range (40). However, both maximum values fell inside the range reported by ILSI database of 53.4–576.2 μg/g dw, while the minimum values fell below (41). Wide ranges of quantification have been reported previously for bound *p*-coumaric acid in maize hybrids. For example, Xu et al. (42) found that conventional hybrids contained a range of 84.79–239.33 μg/g dw, Venkatesh et al. (43) found 232.58–532.51 μg/g dw, Harrigan et al. (44) reported 84.15–259.68 μg/g dw, whereas the values in Classen et al. (45) fluctuated between 29.3 and 219 μg/g dw. Duncan et al. (32) reported that genetically modified hybrids crossed with landraces have ranges between 101.9 and 236.2 μg/g dw. The ILSI database covers most of these ranges and is a reliable alternative for comparison of bound *p*-coumaric acid (41).

Bound *trans*-ferulic acid was the most abundant phenolic acid in both hybrid groups in the current study, and it was present in different ranges in the B73 × Landrace hybrids and B73 × NAM hybrids. NAM hybrids presented a higher variation between the genotypes, but landrace hybrids had a higher variation within the genotypes. This phenolic compound was also the most prominent phenolic in previous studies of maize varieties and landraces (18, 28, 45). Ferulic acid has been largely associated with kernel insect resistance. Early studies established a negative correlation between trans-ferulic acid content in landraces and susceptibility parameters toward maize weevil (46). In a later study comparing phenotypic characteristics of maize kernel in susceptible varieties and resistant maize landraces, *trans*-ferulic acid was one of the most important phenolic acids explaining phenotypic variance in maize weevil resistance (18). The ranges found in the current study fall within the range presented by ILSI database: 291.9–3,885.8 μg/g dw (41). Another study reported extensive variation between hybrids for bound *trans*-ferulic acid, having a four-fold range in values (45). Recently Duncan et al. (32) reported that genetically modified hybrids crossed with landraces have a range between 1,150 and 2,530 μg/g dw. Landraces analyzed for bound *trans*-ferulic acid content, have also show different and wide variations; going from 394 to 1,700 μg/g dw (45), to higher values like 2,521–2,840 μg/g dw (27), or lower like 34–46 μg/g dw (30). It is important to note that in all these reports, only a few landraces were analyzed, compared to the 24 diverse landrace varieties from across the Americas evaluated in the current study. A specific evaluation including over 30 accessions of 14 Chilean maize landraces found lower means and a lower range for bound *trans*-ferulic acid: 126.1–268.5 μg/g dw (47). Also, in multicolored Mexican landraces, the range found was 1,380–1,500 μg/g dw (48). It is worth noticing that in that study, the selection of the material was done according to phenotypic diversity, using color as the parameter of choice, and it was found that orange phenotype contained the highest *trans*-ferulic content. In our study, results strongly suggest that bound *trans*-ferulic acid in landraces has a significant genotype variation and it is wider than the one captured by previous reports. Therefore, the inclusion of landrace materials with a high diversity, like the ones included in this study, is suggested for future diversity studies.

### Effects of Genetic Background and Origin

The effect of genetic background by the type of hybrid was significant for soluble and cell-wall bound *p*-coumaric acid, with the landrace hybrid group having a higher content of the former and a lower content of the latter. This wide variation has been reported with other landraces hybrids (32). In the phenylpropanoid pathway which is responsible for phenolic compound synthesis in plants, *p*-coumaric acid is the first phenolic compound formed. From *p*-coumaric acid, other phenolic compounds are derived like hydroxycinnamic acids, flavones, flavonols, flavanones, and anthocyanins. Normally, *p*-coumaric acid is formed from the deamination and hydroxylation of phenylalanine, but in Graminaceous species like maize, it can also be formed from the deamination of tyrosine (49). This wide diversification of *p*-coumaric acid products makes complicated to find a direct relationship between these compounds, which could explain why *p*-coumaric acid was the only phenolic where a significant difference between hybrid groups was found.

The effect of the geographic origin was significant for both soluble *p*-coumaric acid and *trans*-ferulic acid. This is not surprising as they originated from different ecoregions and these factors show a reflection of the versatility of these materials to adapt to a different cultivation environments, resist diverse types of stress, endosperm characteristics, and the effect over the phenolic compounds that these processes involve (32). Phenolic compounds are active participants of important plant physiology processes that aid in adaptation and resistance; for example, the accumulation of phenolic compounds occurs in plants subjected to mechanical and biological stress (27, 50), changes in *p*-coumaric acid function as signal for different stages of development (51), antimicrobial effects to different types of bacteria (52), modulation of membrane permeability, signal transduction and vesicle trafficking are some of the functions of plant phenolics as signaling molecules (53).

Furthermore, cell wall diferulic acids have been mainly associated with biotic agent resistance in maize varieties (17, 18, 45, 54). A high correlation between maize kernel hardness and maize weevil resistance associated with diferulic acids content and linkages were reported (18). This cross-linking provides resistance by fortifying the pericarp cell wall, increasing physical strength and kernel hardness. Moreover, 8-O-4 DFA has been negatively correlated with maize kernel hardness (55). The wider ranges observed in landrace hybrids could represent the potential of these maize varieties for natural resistance to biological attacks, as they have locally adapted to different environmental challenges.

## Conclusions

The understanding of maize natural and expected diversity in terms of phenolic compounds is a requisite to make a real comparison between traditional landraces and modern inbred varieties. In this study, wide ranges were found in both landrace and NAM hybrids for all phenolic compounds evaluated, showing the diversity of the genotypes for both groups. The only phenolic compound able to detect a difference between modern hybrids and native hybrids was *p*-coumaric acid, in both its soluble and cell-wall bound forms. The effect of origin was significant for soluble *p*-coumaric and *trans*-ferulic acids, denoting the role of phenolic compounds in the ability of maize to endure different environmental conditions. This study also provides further information through the unveiling of maize natural variation in phenolic compounds and serves as a comparison platform. It also suggests that future studies should include higher variation among maize varieties that truly reflect maize natural variation in terms of phenolic compounds and their susceptibility to the environment.

## Data Availability Statement

The raw data supporting the conclusions of this article will be made available by the authors, as per request.

## Author Contributions

MZ-L performed experimental work, and acquired results. SF-G developed and provided the biological material, field design, and reviewed the manuscript. SG-L carried out the data analysis and interpretation, designing the experiment, interpreting results and supervising the overall study. All authors read and approved the final manuscript.

## Conflict of Interest

The authors declare that the research was conducted in the absence of any commercial or financial relationships that could be construed as a potential conflict of interest.
